# CyberKnife Radiosurgery for Extracranial Metastases of Oligodendroglioma: A Clinical Case Report

**DOI:** 10.7759/cureus.51035

**Published:** 2023-12-24

**Authors:** Elaheh Shaghaghian, David J Park, Kelly H Yoo, Antonio Meola, Steven D Chang

**Affiliations:** 1 Department of Neurosurgery, Stanford University, School of Medicine, Palo Alto, USA

**Keywords:** radio surgery, stereotactic radio surgery (srs), extra cranial metastasis, oligodendroglioma, stereotactic radiosurgery (cyberknife®)

## Abstract

Oligodendroglioma is an uncommon glial tumor known for its extremely rare tendency to metastasize to extracranial areas, particularly to the spinal region.

We present a rare case of oligodendroglioma that metastasizes to the spinal cord 14 years after resection of the initial tumor. Furthermore, a systematic review of the relevant literature is conducted following Preferred Reporting Items for Systematic Reviews and Meta-Analyses (PRISMA) guidelines, encompassing oligodendroglioma cases with extracranial metastases. Our PRISMA-guided systematic review fills a critical knowledge gap in neurosurgery by consolidating scattered data on oligodendroglioma metastases, offering pivotal insights for clinical practice and future research.

A 50-year-old male patient exhibited severe headaches and dizziness, with MRI findings revealing a significant brain mass suggestive of oligodendroglioma. Consequently, the patient underwent a craniotomy procedure. After 14 years, the patient presented with weakness in both lower extremities and bladder as well as bowel dysfunction. A lumbosacral MRI of the patient revealed two intradural enhancing masses in the lumbosacral spine. Surgical resection was performed, and the characteristics were identical to those of the primary tumor.

The systemic review encompassed 52 articles, covering 67 cases of extracranial metastases from oligodendroglioma. Only three cases in the literature review fulfilled the inclusion criteria, demonstrating the required molecular genetic profiles of isocitrate dehydrogenase​​​ (IDH)-mutation and chromosome 1p/19q-codeletion. The inclusion criteria encompassed cases of oligodendroglioma with confirmed extracranial metastases, focusing on those with documented molecular genetic profiles indicating IDH-mutation and 1p/19q-codeletion. Our emphasis was on identifying cases with this specific genetic profile to ensure consistency and relevance in the literature review. Interestingly, our case was the first to exhibit intradural spinal metastases, while the other cases involved metastasis to the spinal bone marrow.

Our case and literature review demonstrate that oligodendrogliomas, although exceptionally rare, can metastasize not only to the extracranial area but also to the spinal cord. To improve survival in oligodendroglioma cases, it is recommended to implement regular radiological screening and monitoring to enable early detection and treatment of extracranial metastases.

## Introduction

Oligodendroglioma is a rare disease that constitutes five percent of all malignant adult primary brain tumors, with an annual incidence rate of one to two cases per million [1,2]. Oligodendrogliomas are most commonly located in the frontal lobe of the brain [3,4]. The World Health Organization (WHO) classification, published in May 2021, has incorporated histologic characteristics and genetic alterations (isocitrate dehydrogenase​​​ (IDH)-mutation and chromosome 1p/19q-codeletion) to categorize oligodendrogliomas into grade 2 and grade 3. The diagnosis of oligodendroglioma now relies on genetic alterations, and the term "anaplastic oligodendroglioma" is no longer used in the WHO 2021 classification. Instead, they are now referred to as grades 2 and 3 oligodendrogliomas, respectively [1,5,6].

Oligodendrogliomas metastasize through three primary routes: local infiltration, seeding via the cerebrospinal fluid (CSF), and lymphatic dissemination. Extracranial metastases, notably to sites such as bone, lymph nodes, and bone marrow, remain exceptionally rare in oligodendrogliomas [5,7-10]. This study presents a case of a patient with oligodendroglioma grade 3 who developed spinal metastases 14 years after the initial diagnosis and a systematic review of cases of oligodendroglioma with extracranial metastases. Additionally, we performed a systematic review following standard Preferred Reporting Items for Systematic Reviews and Meta-Analyses (PRISMA) guidelines to identify cases of oligodendroglioma with extracranial metastases reported from 1951 to 2023.

## Case presentation

We present a rare case of oligodendroglioma in which the tumor metastases to the spinal cord 14 years after resection of the initial brain tumor. Additionally, we performed a systematic review following standard PRISMA guidelines to identify cases of oligodendroglioma with extracranial metastases reported from 1951 to 2023.

**Figure 1:**
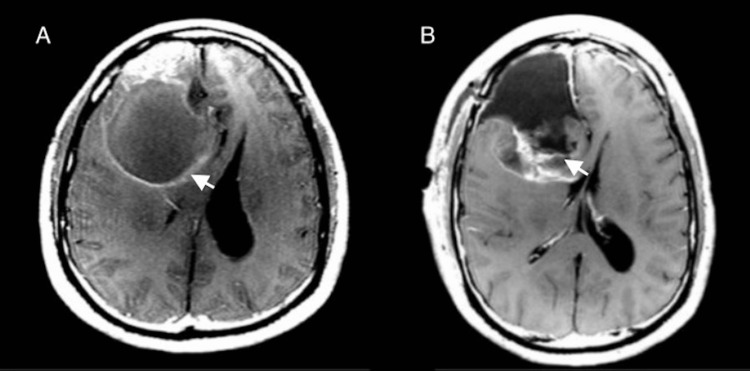
Right frontal oligodendroglioma A: Initial preoperative axial postcontrast T1-weighted MRI revealed a large right frontal lobe mass associated with focal calcifications and possible blood products suggestive of oligodendroglioma with a midline shift to the left. B: Postoperative MRI after initial surgery presents gross total resection of the tumor with a persistent mass effect 9 mm leftward midline shift.

A 50-year-old male patient went to the emergency department, displaying severe headaches and dizziness. His initial brain magnetic resonance imaging (MRI) demonstrated a large right frontal lobe mass associated with focal calcifications and possible blood products suggestive of oligodendroglioma with mass effect. It revealed a 1.3 cm midline shift to the left (Figure 1A). Then, the patient underwent the first gross total resection of the right frontal tumor (Figure 1B).

**Figure 2:**
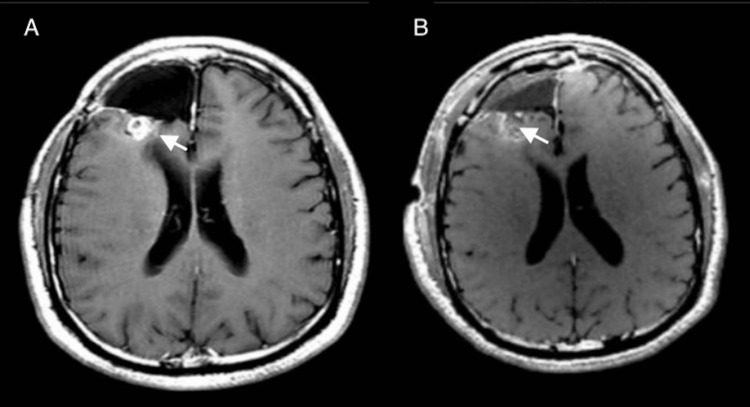
Two-year follow-up of right frontal tumor A: Postcontrast axial T1-weighted MRI two years after the initial surgery presents a focal area of nodular enhancement, suspicious of tumor recurrence. B: Postoperative MRI reveals postoperative changes compatible with the resection of the right frontal recurrent tumor.

Histopathological examination confirmed the diagnosis of oligodendroglioma, IDH1-mutant, and 1p/19q-codeleted (WHO grade 3). The patient was treated with 12 cycles of temozolomide (TMZ). Two years later, the brain tumor recurred. The brain MRI showed a focal area of nodular enhancement with increased blood volume along the posterior area of the prior resection margin, suggestive of tumor progression (Figure 2A). The patient underwent a second craniotomy and gross total resection (Figure 2B).

**Figure 3:**
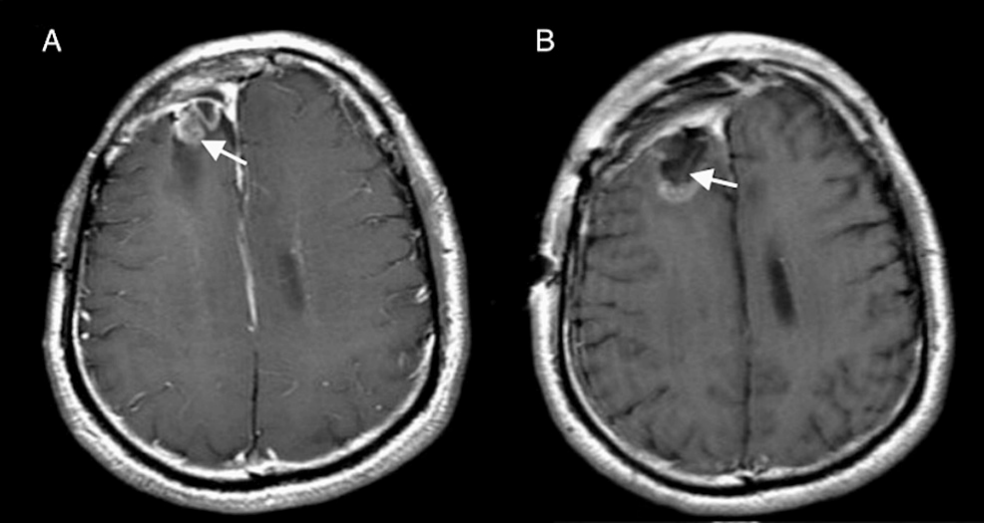
Three-year follow-up: recurrent right frontal tumor A: Preoperative axial postcontrast T1-weighted MRI, three years after initial surgery, demonstrates a 6 mm enhancing nodular focus above the initial resection cavity in the right frontal lobe suggestive of tumor recurrence. B: Postoperative MRI presents gross total resection of the enhancing nodule.

The patient received lomustine (CCNU) after surgery for five cycles. After one year, he underwent the third craniotomy for a new 6 mm focus above the initial resection cavity in the right frontal lobe, suggesting tumor recurrence (Figure 3A-B).

**Figure 4:**
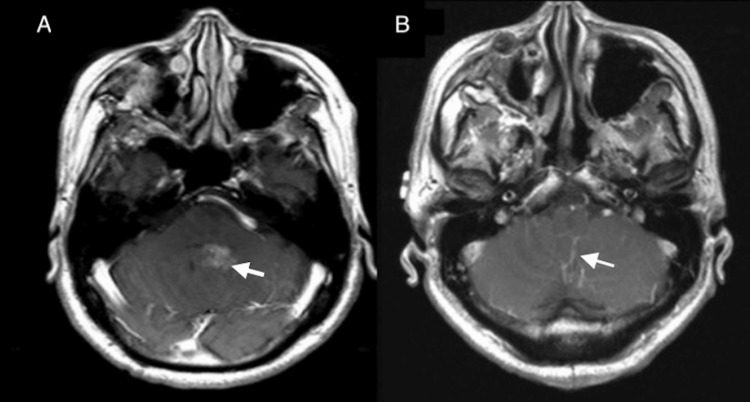
Ten-year follow-up showing mass in the fourth ventricle A: Preoperative MRI, ten years after initial surgery, presents an enhancing mass in the fourth ventricle. B: Postoperative axial postcontrast T1-weighted MRI after suboccipital craniotomy demonstrates gross total resection of the mass in the fourth ventricle.

Ten years after the initial surgery, the MRI revealed an interval increase in the size of nodules extending into the fourth ventricle concerning tumor progression (Figure 4A). The patient underwent a suboccipital craniotomy for resection of the mass along the dorsal aspect of the fourth ventricle (Figure 4B).

**Figure 5:**
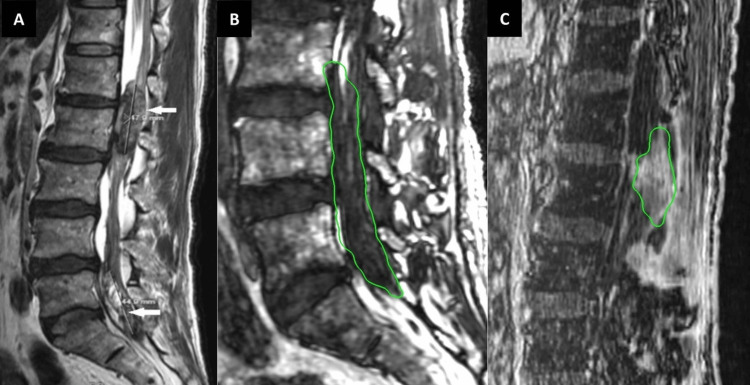
Fourteen-year follow-up: CyberKnife radiosurgery treatment for spinal metastases secondary to oligodendroglioma A: Preoperative sagittal T2-weighted lumbosacral spine MRI 14 years after the initial surgery presents two intradural enhancing masses (indicated by white arrows) in the lumbosacral spine. The rostral mass extends from L1 to L2, and the caudal mass extends from the L4-L5 disc space through the S1 spine. B: After 12 days of the last lumbosacral surgery, CK SRS was planned for the L4-S1 lesion. A marginal dose of 25.5 Gy was prescribed to the 83% isodose line. The maximum dose was 30.91 Gy (target contoured in green line). C: Two months after the last CK SRS treatment, another CK SRS was planned for recurrent L1-L3 lesions. This lesion was treated with a marginal dose of 25.5 Gy to the 80% isodose line. The maximum dose was 31.88 Gy. The gross tumor volume and clinical target volume were the same for these intradural lesions (target contoured in green line). CK SRS - CyberKnife stereotactic radiosurgery

Pathology results confirmed a recurrent oligodendroglioma, IDH1-mutant, and 1p/19q-codeleted (WHO grade 3). Then, he started bevacizumab. Fourteen years later, during which he was carefully under follow-up with a brain MRI, the patient returned complaining of both lower extremities weakness, although a specific scale was not explicitly documented. He had a sensory loss of both lower extremities with loss of bladder and bowel function. MRI revealed two intradural enhancing masses in the spine. The first extended from L1 to L2, and the second one extended from L4-L5 disc space through S1, where it completely effaced the anterior thecal sac with the encasement of numerous nerve roots (Figure 5A).

The patient underwent L1-L3 lumbar laminectomy for tumor resection. The histological findings revealed metastatic oligodendroglioma, IDH1-mutant, and 1p/19q-codeleted (WHO grade 3). Twelve days after the L1-L3 laminectomy, CyberKnife (CK; Accuray, Inc., Sunnyvale, CA) stereotactic radiosurgery (SRS) was performed for the L4-S1 lesion with a marginal dose of 25.5 Gy in three fractions to 83% isodose line (Figure 5B). Two months after the first SRS treatment, the patient underwent CK SRS to recurrent L1-L3 lesion, which was previously resected sub-totally. A marginal dose of 25.5 Gy in three fractions to 80% isodose line was delivered (Figure 5C). After CK SRS for both lumbar lesions, the patient was clinically and radiologically stable for 12 months until he had a fall.

The last brain MRI, which was taken when he had a fall 15 years after the initial diagnosis, showed continued tumor progression with the right mesial temporal, right thalamic, corpus callosum, and intraventricular/periventricular area. Bevacizumab infusion was restarted every three weeks for palliation. Treatment options were discussed. The patient decided on do-not-resuscitate (DNR) and do-not-intubate (DNI) and wished to switch to comfort care. He is being followed up with brain, thoracic, and lumbar spine MRI and treated with bevacizumab infusions.

## Discussion

**Figure 6:**
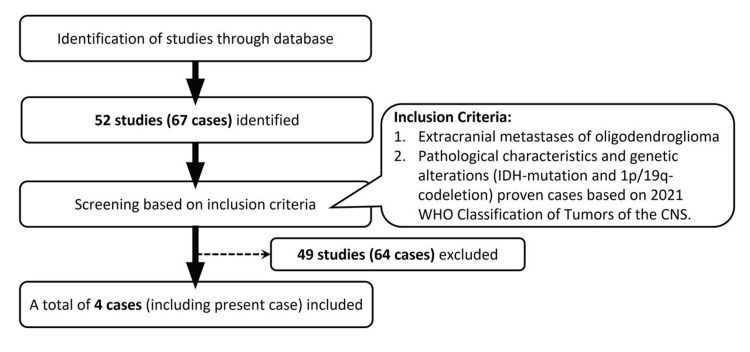
Systematic review of extracranial metastases of oligodendroglioma Diagram of the PRISMA workflow with inclusion criteria for the systematic review of extracranial metastases of oligodendroglioma. A total of 52 studies with 67 cases were identified from 1951 to 2023. Based on further review of the inclusion criteria, only four cases (including the present case) had extracranial metastases of oligodendrogliomas with molecular genetic profiles of IDH-mutation and 1p/19q-codeletion proven in the record. PRISMA - Preferred Reporting Items for Systematic Reviews and Meta-Analyses; WHO - World Health Organization; IDH-mutation - isocitrate dehydrogenase-mutation; 1p/19q codeletion - chromosome 1p/19q codeletion; CNS - central nervous system

To identify cases of oligodendroglioma with extracranial metastases, we conducted a systematic review following PRISMA guidelines. A PubMed search using specific keywords yielded 52 studies reporting a total of 67 extracranial tumors of oligodendroglioma from 1951 to 2023 (see Figure 6). By applying the inclusion criteria of IDH-mutation and 1p/19q-codeletion confirmed through pathological examination, only four cases, including our present case, fulfilled the criteria. Our patient represents an exceptionally rare case of oligodendroglioma with pathologically confirmed IDH1-mutation and 1p/19q-codeletion (WHO grade 3) that metastasized to the spinal cord in the intradural region. 

However, only four cases, including our present case, fulfilled the criteria of oligodendrogliomas with molecular genetic profiles of IDH-mutation and 1p/19q-codeletion (WHO 2021) accompanied by extracranial metastases (Table 1) [3,11,12]. Among these cases, three had metastases to the spinal bone marrow, while our case exhibited metastasis solely to the spinal cord. The gender distribution was two female and two male patients, with ages at diagnosis ranging from 35 to 71 years. Three cases were classified as grade 3 oligodendroglioma, while one case was grade 2. The initial tumor location in three cases was the right frontal lobe, while one case originated from the right temporoparietal area. In the remaining three cases, distant metastasis occurred in the bone marrow of the spine. The time interval from the initial diagnosis to distant metastasis varied among patients, ranging from 20 to 180 months. 

**Table 1:** Clinical case details A systematic review of cases with extracranial metastases of an IDH-mutant and 1p/19q co-deleted oligodendroglioma (either grade 2 or 3 based on 2021 WHO classification of CNS tumors). IDH-mutation - isocitrate dehydrogenase-mutation; 1p/19q codeletion - chromosome 1p/19q codeletion; CNS - central nervous system

Patient No.	Authors (year)	Year	Gender	Age at diagnosis	Pathology (WHO grade)	Location of the initial tumor	Location of the distant metastasis	Treatment	Time of distant metastasis from the initial diagnosis (months)	History of previous surgery	History of radiation
1	Burgy et al. [3]	2019	M	36	Oligodendroglioma (grade 3)	Right temporoparietal lobe	Bone marrow of the spine, pelvis, rib	Surgery and chemotherapy	29	Yes	No
2	Demeulenaere et al. [11]	2016	F	35	Oligodendroglioma (grade 3)	Right frontal lobe	Bone marrow of the spine	Surgery and chemotherapy and stereotactic radiation therapy (Novalis; 60 Gy)	180	Yes	Yes
3	Kiko et al. [12]	2018	F	71	Oligodendroglioma (grade 2)	Right frontal lobe	Bone marrow of the spine, mandibular bone	Surgery, chemotherapy and radiotherapy	20	Yes	Yes
4	Present case	2023	M	50	Oligodendroglioma (grade 3)	Right frontal lobe	Intradural spine	Surgery and chemotherapy and stereotactic radiosurgery	168	Yes	Yes

**Figure 7:**
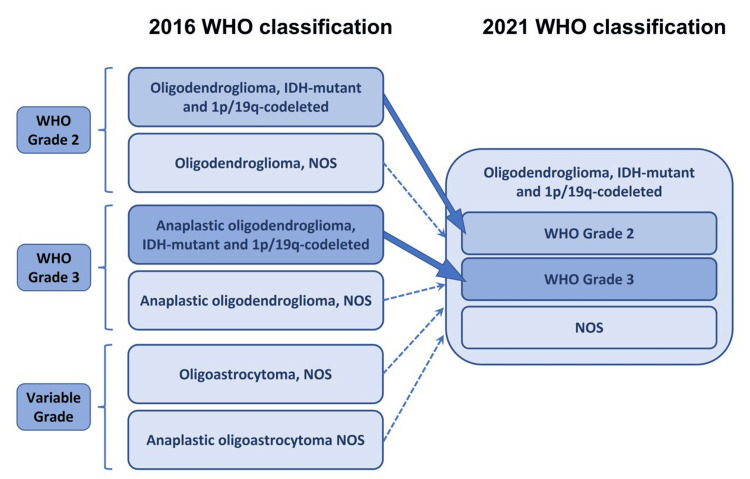
WHO classification of oligodendroglioma Schematic figure presenting how the classification of oligodendroglioma changed from the 2016 WHO classification to the 2021 WHO classification. Solid lines denote higher correlations between the two classifications, and dotted lines denote lower correlations. WHO: World Health Organization; IDH-mutant - isocitrate dehydrogenase-mutant; NOS - not otherwise specified The image is created by the authors.

According to the updated 2021 WHO classification for central nervous system (CNS) tumors, the diagnosis of all oligodendroglioma requires the co-occurrence of IDH-mutation and 1p/19q-codeletion (Figure 7).

To the best of our knowledge, this is the first documented case of intradural spinal metastases in a patient with pathologically confirmed IDH1-mutant and 1p/19q-codeleted oligodendroglioma. The rarity of this case can be attributed to two key aspects. Firstly, extracranial metastases of oligodendroglioma are very rare, especially in the spine. Moreover, while occurrence in the spinal column has been described in three other cases, it has never been reported in the intradural space. Secondly, despite high-grade oligodendrogliomas typically having a poor short-term prognosis, the metastasis in this case occurred exceptionally late, 14 years after the initial brain tumor presentation. This underscores the importance of comprehensive craniospinal radiological follow-up in long-term survivors.

Previous studies by Demeulenaere et al. [11] and Kiko et al. [12] described cases where the frontal lobe was the initial site of the brain tumor, similar to our present case. However, the exact route of metastatic spread remains uncertain. Possible mechanisms include local infiltration, CSF seeding, or lymphatic invasion as tumor cells traverse the dura mater or leptomeninges [3]. Irrespective of the mechanism, the high incidence of intracranial tumor recurrences and prolonged survival may have provided an opportunity and time for the development and symptomatic manifestation of spinal metastasis. These factors should have prompted a comprehensive radiological evaluation of the entire central nervous system before the clinical presentation of spinal metastasis.

While high-grade gliomas have five-year survival rates of five percent, low-grade gliomas have rates as high as 80% [13]. Our present case experienced an exceptional interval of 168 months between the initial diagnosis and extracranial metastasis. The genetic loss of 1p/19q chromosomes is associated with high sensitivity to chemotherapy [14 ]. The presented case, similar to the previously reported cases, received multiple cycles of chemotherapy with TMZ or peplomycin, ACNU (nimustine) and vincristine (PAV)/procarbazine, CCNU (lomustine) and vincristine (PCV), which may have contributed to the extended survival.

SRS represents a minimally invasive adjuvant treatment for patients with residual or recurring oligodendrogliomas. It could also be taken into account as an alternative to primary excision in cases of small-volume tumors found in the eloquent area of the brain [15]. Our presented case demonstrates good local tumor control of CK SRS to extracranial metastatic oligodendroglioma. Multi-centric larger studies on the use of SRS for IDH-mutant 1p/19q-codeleted oligodendroglioma with extracranial metastasis would be helpful for confirming its safety and effectiveness. 

Our study has several limitations. Firstly, during the literature review, many of the case reports lacked sufficient information on histologic and genetic profiling, which hindered the precise diagnosis of oligodendroglioma. Additionally, due to the limited number of cases available in the literature, we were unable to conduct significant statistical analysis. The small sample size restricts the generalizability of our findings. This lack of comprehensive data may have affected the completeness of our report.

## Conclusions

In conclusion, extracranial metastases in oligodendrogliomas are rare occurrences. Patients with a history of multiple prior intracranial surgeries and extended survival may be predisposed to extracranial metastasis, with a significant time interval between the initial diagnosis and metastasis. In this report, we present an exceptional case of a patient who developed intradural spinal metastases secondary to IDH1-mutant and 1p/19q-codeleted oligodendroglioma 14 years after the initial gross total resection of the intracranial tumor. We emphasize the importance of radiological surveillance of the entire central nervous system to facilitate early treatment before metastasis becomes symptomatic. Also, SRS provides reasonable local tumor control for intradural metastases secondary to oligodendroglioma. The study underscores the importance of extended follow-up data and larger cohort studies to validate and broaden these initial observations. Further research investigating alternative therapies and inclusive methodologies is pivotal in advancing the understanding and management of oligodendroglioma metastases.

## References

[1] Wu Y, Liu B, Qu L, Tao H (2011). Extracranial skeletal metastasis in anaplastic oligodendroglioma: case report and review of the literature. J Int Med Res.

[2] Kinslow CJ, Garton AL, Rae AI (2019). Extent of resection and survival for oligodendroglioma: a U.S. population-based study. J Neurooncol.

[3] Burgy M, Chenard MP, Noël G, Bourahla K, Schott R (2019). Bone metastases from a 1p/19q codeleted and IDH1-mutant anaplastic oligodendroglioma: a case report. J Med Case Rep.

[4] Liu S, Liu X, Xiao Y, Chen S, Zhuang W (2019). Prognostic factors associated with survival in patients with anaplastic oligodendroglioma. PLoS One.

[5] Noshita N, Mashiyama S, Fukawa O, Asano S, Watanabe M, Tominaga T (2010). Extracranial metastasis of anaplastic oligodendroglioma with 1p19q loss of heterozygosity - case report. Neurol Med Chir.

[6] Osborn AG, Louis DN, Poussaint TY, Linscott LL, Salzman KL (2022). The 2021 World Health Organization classification of tumors of the central nervous system: what neuroradiologists need to know. AJNR Am J Neuroradiol.

[7] Kim JG, Park CO, Hyun DK, Ha YS (2003). Spinal epidural metastasis of cerebral oligodendroglioma. Yonsei Med J.

[8] Wu L, Ou Y, Liu B, Liu W (2019). Scalp metastasis of anaplastic oligodendroglioma. World Neurosurg.

[9] Volavsek M, Lamovec J, Popović M (2009). Extraneural metastases of anaplastic oligodendroglial tumors. Pathol Res Pract.

[10] Cao L, Rong P, Zhu G, Xu A, Chen S (2021). Clinical characteristics and overall survival prognostic nomogram for oligodendroglioma: a surveillance, epidemiology, and end results population-based analysis. World Neurosurg.

[11] Demeulenaere M, Duerinck J, DU Four S, Fostier K, Michotte A, Neyns B (2016). Bone marrow metastases from a 1p/19q co-deleted oligodendroglioma - a case report. Anticancer Res.

[12] Kiko K, Suyama K, Yamamoto K (2018). A case of bone marrow metastasis of oligodendroglioma with IDH mutation and 1p/19q codeletion (in Japanese). No Shinkei Geka.

[13] Whitfield BT, Huse JT (2022). Classification of adult-type diffuse gliomas: Impact of the World Health Organization 2021 update. Brain Pathol.

[14] Yao J, Hagiwara A, Raymond C (2020). Human IDH mutant 1p/19q co-deleted gliomas have low tumor acidity as evidenced by molecular MRI and PET: a retrospective study. Sci Rep.

[15] Kano H, Niranjan A, Khan A, Flickinger JC, Kondziolka D, Lieberman F, Lunsford LD (2009). Does radiosurgery have a role in the management of oligodendrogliomas?. J Neurosurg.

